# Generation of primitive neural stem cells from human fibroblasts using a defined set of factors

**DOI:** 10.1242/bio.013151

**Published:** 2015-10-21

**Authors:** Takumi Miura, Tohru Sugawara, Atsushi Fukuda, Ryo Tamoto, Tomoyuki Kawasaki, Akihiro Umezawa, Hidenori Akutsu

**Affiliations:** 1 Department of Reproductive Biology, National Center for Child Health and Development, Tokyo 157-8535, Japan; 2 Department of Stem Cell Research, Fukushima Medical University, Fukushima 960-1295, Japan

**Keywords:** Neural stem cell, Reprogramming, Stem cells

## Abstract

In mice, leukemia inhibitory factor (LIF)-dependent primitive neural stem cells (NSCs) have a higher neurogenic potential than bFGF-dependent definitive NSCs. Therefore, expandable primitive NSCs are required for research and for the development of therapeutic strategies for neurological diseases. There is a dearth of suitable techniques for the generation of human long-term expandable primitive NSCs. Here, we have described a method for the conversion of human fibroblasts to LIF-dependent primitive NSCs using a strategy based on techniques for the generation of induced pluripotent stem cells (iPSCs). These LIF-dependent induced NSCs (LD-iNSCs) can be expanded for >100 passages. Long-term cultured LD-iNSCs demonstrated multipotent neural differentiation potential and could generate motor neurons and dopaminergic neurons, as well as astrocytes and oligodendrocytes, indicating a high level of plasticity. Furthermore, LD-iNSCs easily reverted to human iPSCs, indicating that LD-iNSCs are in an intermediate iPSC state. This method may facilitate the generation of patient-specific human neurons for studies and treatment of neurodegenerative diseases.

## Abstract (precis)

**Summary:** A novel method for the generation of self-renewable, multipotent and neural lineage-restricted LIF-dependent induced primitive neural stem cells (LD-iNSCs) from human fibroblasts with the potential to facilitate human neuronal studies.

## INTRODUCTION

Yamanaka's discovery of induced pluripotent stem cells (iPSCs) was a breakthrough innovation in the field of cellular reprogramming, which is the conversion of differentiated cells into an undifferentiated state (Takahashi et al., 2007; Takahashi and Yamanaka, 2006). Concepts emerging from iPSC technology have provided valuable opportunities for the expansion of research on transdifferentiation, or the irreversible conversion of a specific cell type to another via forced expression of lineage-related transcription factors (Caiazzo et al., 2011; Efe et al., 2011; Huang et al., 2011; Ieda et al., 2010; Pang et al., 2011; Pereira et al., 2013; Pfisterer et al., 2011; Vierbuchen et al., 2010; Yoo et al., 2011). Therefore, cells converted into a desired cell type could be useful as a new tool for the discovery of therapeutic targets and pathogenic processes. Nonetheless, because most of the converted cells are terminally differentiated, their proliferation potential is much lower compared to that of multipotent stem cells, suggesting that direct lineage conversion technology has limitations in drug screening or autologous cell therapy. To address this issue, direct conversion of somatic cells to lineage-committed multipotent stem cells might be an attractive approach for indirect generation of desired cell types. This indirect approach would allow for the production of sufficient amounts of cells for basic research, drug screening, disease modeling, or cell therapy.

Some researchers have recently reported that multipotent neural stem/progenitor cells (NSCs) can be directly induced from fibroblasts using neural progenitor-specific transcription factors or the Yamanaka factors as reprogramming agents (Han et al., 2012; Kim et al., 2011; Lujan et al., 2012; Ring et al., 2012; Thier et al., 2012; Wang et al., 2013). These induced neural stem/progenitor cells (iNSCs) are cultured in a standard neural stem cell medium supplemented with basic fibroblast growth factor (bFGF) and epidermal growth factor (EGF). Under these culture conditions, the transition of NSCs into a glia-restricted precursor state occurs over time (Qian et al., 2000). These observations suggest that the neurogenic potential of bFGF-dependent definitive NSCs may be diminished by environmental factors. On the other hand, the earliest mammalian NSCs (primitive NSCs) can differentiate from pluripotent embryonic stem cells (ESCs). Primitive NSCs have more diverse developmental opportunities in neural lineages than definitive NSCs. These primitive NSCs emerge in response to leukemia inhibitory factor (LIF), have distinct growth factor requirements, express neural precursor markers, generate neurons and glia *in vitro*, and have a neural and non-neural lineage potential *in vivo* (Li et al., 2011; Tropepe et al., 2001). Recently, a combination of Yamanaka factors and small molecules was shown to directly convert human fibroblasts into LIF-dependent neural progenitors (NPs), using a nonintegrating method (Lu et al., 2013). Nonetheless, these LIF-dependent induced NPs (iNPs) gradually lose their ability to be re-specified to other fates during long-term passaging (>20 passages). This phenomenon indicates that persistent expression of exogenous reprogramming factors may be required for the maintenance of neurogenic potential in iNPs.

In this study, we present a novel method for the generation of self-renewable, multipotent, and neural lineage-restricted LIF-dependent induced primitive NSCs (LD-iNSCs) from human fibroblasts. These LD-iNSCs exhibit a characteristic morphology, gene expression patterns, and growth rate, as well as a predictable *in vitro* differentiation potential. Specifically, these stable, expandable LD-iNSC clones possess the plasticity of NSCs and can differentiate into neurons (motor neurons and dopaminergic neurons), astrocytes, and oligodendrocytes. Furthermore, LD-iNSCs retain high neurogenic potential even after long-term expansion and repeated passaging in the presence of LIF, CHIR99021 and PD0325901. Our results demonstrate that expandable human primitive NSCs can be directly generated from somatic cells using the same factors known to reprogram cells for pluripotency, under special conditions.

## RESULTS

### LD-iNSCs are directly induced from human fibroblasts by five factors with two inhibitors (2i)

**Fig. 1.**
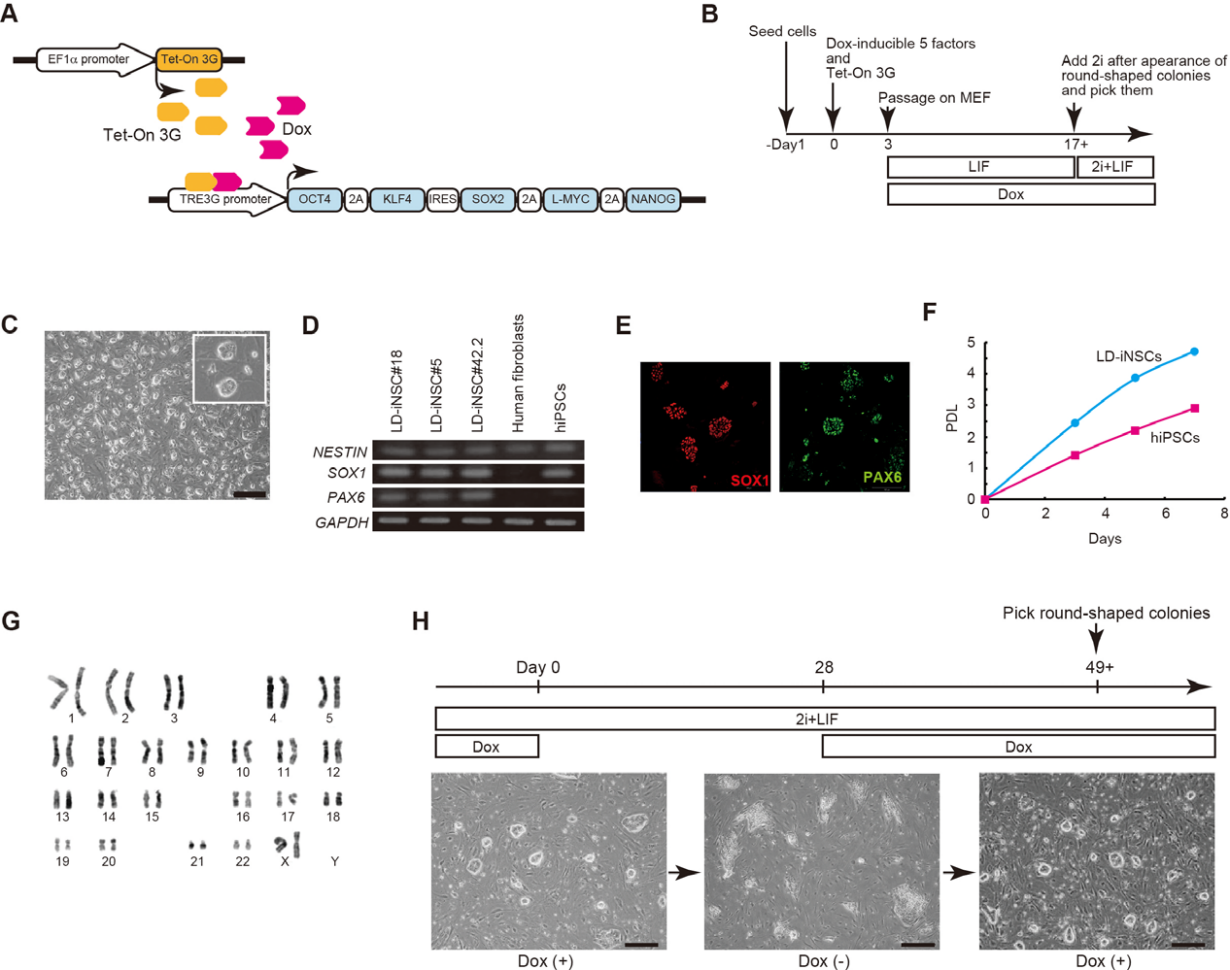
**Direct reprogramming of fibroblasts into LD-iNSCs.** (A) Schematic representation of the doxycycline (Dox)-inducible lentiviral polycistronic vectors. The vector consists of a polycistronic gene that contains the human cDNA sequences of *OCT4*, *KLF4*, *SOX2*, *L-MYC* and *NANOG*, bound by self-cleaving 2A elements (2A) and IRES, and driven by the TRE3G promoter, which is activated by the binding of the Tet-On 3G transactivator in the presence of Dox in a culture medium. (B) Schematic of the experimental setup and strategy for the generation of LIF-dependent induced neural progenitors (LD-iNSCs). (C) Colony morphology of LD-iNSCs exhibited a tightly packed structure similar to primary neural progenitors. (D) RT-PCR showed that LD-iNSCs expressed the neural progenitor markers *NESTIN*, *SOX1* and *PAX6*. *GAPDH* was used as an internal control. RNA was extracted from three LD-iNSC clones (LD-iNSC#5, LD-iNSC#18 and LD-iNSC#42.2), uninfected parent fibroblasts (negative control), and from human iPSCs. (E) LD-iNSCs cultured on feeder cells in the presence of 2i/LIF tested positive for SOX1 (red) and PAX6 (green). (F) The population doubling level (PDL) of LD-iNSCs in comparison with human iPSCs. (G) A representative karyotype of LD-iNSCs at passage 28. (H) Ectopic factor dependence of LD-iNSCs. Upon doxycycline withdrawal, LD-iNSC colony morphology was lost, and the cells assumed a square morphology. The subsequent addition of Dox reversibly produced LD-iNSCs. Days of differentiation or reprogramming are indicated. Scale bars are 500 μm (C and H).

A recent report indicated that LIF-dependent human primitive neural stem cells differentiate from hiPSCs in a chemically defined medium containing LIF and two inhibitors (MEK1/MEK2 inhibitor PD0325901 and GSK-3β inhibitor CHIR99021), collectively termed 2i (Hirano et al., 2012). In this study, we generated LIF-dependent human iNSCs from adult tissues using the doxycycline (Dox)-inducible lentiviral vector transduction method: human fibroblasts were infected with lentivirus-containing reprogramming factors. Our protocol for the generation of human iNSCs in the presence of LIF is shown in Fig. 1A and B. We first analyzed the ability of the reprogramming factors to directly reprogram human fibroblasts to NSCs. To efficiently introduce reprogramming factors into human fibroblasts (endometrial fibrotic stromal cells), we generated polycistronic viral vectors that would express either four (*OCT4*, *KLF4*, *SOX2* and *L-MYC*) or five (*OCT4*, *KLF4*, *SOX2*, *L-MYC* and *NANOG*) reprogramming genes from a single promoter using 2A peptides and internal ribosomal entry sites (IRES). The generation efficiency of iNSCs was assessed using an assay for alkaline phosphatase-positive colonies, based on a previous report that LIF-dependent primitive NSCs derived from hESCs are positive for alkaline phosphatase (Li et al., 2011). As shown in Fig. S1, endogenous alkaline phosphatase-positive colonies effectively emerged after treatment with the five reprogramming factors, but not with only four factors. Reprogramming of human fibroblasts in the presence of LIF using this lentiviral vector (five reprogramming factors) resulted in the formation of round colonies, starting approximately two weeks after the addition of Dox. Subsequently, these round colonies were picked and maintained in the presence of LIF, Dox and 2i, which are known to promote reprogramming (Fig. 1B). These clones were expanded for further clonal analysis.

We eventually established nine individual colonies. These neuroepithelium-like cells grew as tightly packed, bright, round-shaped colonies on feeder layers (Fig. 1C), and were confirmed based on their expression of neural stem cell markers (NESTIN, SOX1 and PAX6; Fig. 1D,E, Fig. S2A). We refer to these cells as human LIF-dependent induced neural stem cells (human LD-iNSCs). Three LD-iNSC clones (#5, #18 and #42.2) were selected for further analysis. We verified the integration of copies of the lentiviral vector into the genome of these LD-iNSCs (clones #5, #18, #42.2) by Southern blotting for two independent sites in the lentiviral vector. Southern blotting confirmed that the three cell lines (#5, #18 and #42.2) were independent clones (data not shown). In addition, short tandem repeat (STR) analysis of LD-iNSCs confirmed that these cells were derivatives of the parental human fibroblasts (Table S1).

We further characterized these established LD-iNSC clones. These cells grew at a much higher proliferation rate than hiPSCs, with a doubling time of approximately 23–25 h (Fig. 1F). These cells have now been expanded for more than 100 passages in the presence of 2i/LIF/Dox, thus generating a large number of cells. Moreover, the LD-iNSC lines retained a normal karyotype (2n=46, XX; Fig. 1G) at passage 28.

Next, we tested whether growth of LD-iNSCs in the presence of 2i/LIF could reactivate the inactive X chromosome, based on previous reports that the inactive X chromosome in hiPSCs is reactivated in the presence of 2i/LIF (Hanna et al., 2010; Wang et al., 2011). However, this may be specific to the culture conditions of hiPSCs. Therefore, we analyzed the effect of 2i/LIF conditions on X inactivation in LD-iNSCs. Nonetheless, as shown in Fig. S2B and C, LD-iNSCs grown in a medium containing 2i/LIF expressed an XIST cloud in all cells, which is indicative of the continued X chromosome inactivation.

We then tested whether LD-iNSCs could be stably propagated in the absence of the five ectopic reprogramming factors (OCT4, KLF4, SOX2, L-MYC and NANOG). As shown in Fig. 1H, the withdrawal of Dox for four weeks resulted in a gradual loss of the rounded NSC-like colony morphology, with all cells adopting a small flat epithelial cell-like appearance. These LD-iNSCs underwent morphological changes into neurons in response to longer-term withdrawal of Dox (Fig. S2D). This result suggested the need for some ectopic reprogramming factors for the maintenance of LD-iNSCs derived from human fibroblasts. On the other hand, round colony morphology was observed approximately three weeks after the re-addition of Dox (Fig. 1H), suggesting that LD-iNSCs depend on the expression of exogenous reprograming factors. Such strong dependence on the constitutive ectopic expression of reprograming factors during cell maintenance indicates that the LD-iNSCs were reprogrammed to a metastable state.

### Characterization of LD-iNSCs

**Fig. 2.**
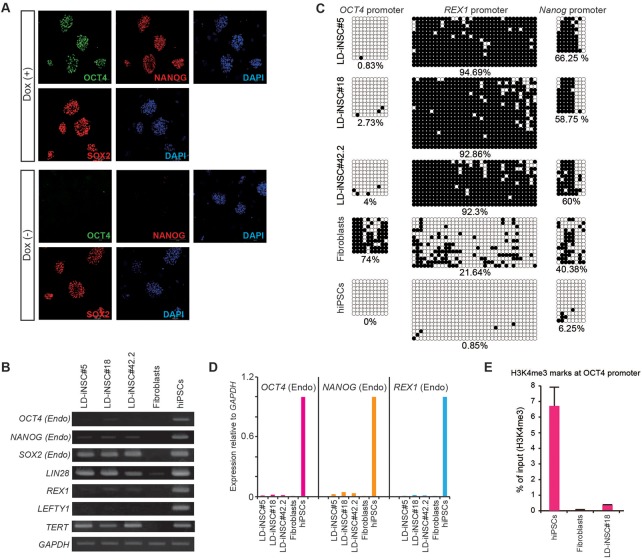
**Characterization of LD-iNSCs.** (A) Immunostaining for OCT4, SOX2 and NANOG in LD-iNSCs with (top) or without (bottom) Dox treatment. Only SOX2 expression was detected in LD-iNSCs without Dox (bottom). Cell nuclei were visualized with DAPI. (B) RT-PCR showed that LD-iNSCs expressed markers of pluripotency, such as endogenous *OCT4*, *NANOG*, *SOX2*, *LIN28*, *REX1*, *LEFTY1* and *TERT*. *GAPDH* served as the internal control. RNA was extracted from three LD-iNSC clones (LD-iNSC#5, LD-iNSC#18 and LD-iNSC#42.2), uninfected parent fibroblasts (negative control), and from human iPSCs (positive control). (C) DNA methylation status of CpG islands in the *OCT4*, *REX1* and *NANOG* promoter regions was assessed by bisulfite sequencing PCR. Open circles indicate unmethylated, and filled circles indicate methylated, CpG dinucleotides. Representative sequences from three LD-iNSC clones (LD-iNSC#5, LD-iNSC#18 and LD-iNSC#42.2), uninfected parent fibroblasts (negative control) and human iPSCs (positive control) are shown. The percentage of CpG methylation in each CpG island within the respective cell line is indicated. (D) Expression levels of pluripotent marker genes (*OCT4*, *REX1* and *NANOG*) selected in (C). All data are normalized to hiPSCs (positive control), whose expression was assumed to be 1.0 for genes in other cell lines. (E) ChIP-qPCR analysis of the presence of histone 3 lysine 4 marker in the promoter region of the pluripotency gene *OCT4* in LD-iNSC#18, uninfected parent fibroblasts (negative control), and human iPSCs (positive control). Error bars are mean±s.e.m., *n*=3.

Pluripotency markers (OCT4, SOX2 and NANOG) were actively expressed in our LD-iNSCs cultured with Dox (Fig. 2A). On the other hand, when Dox was withdrawn for three days, SOX2 was detected in LD-iNSCs but neither OCT4 nor NANOG were detected (Fig. 2A). Consequently, SOX2 must have been expressed endogenously in LD-iNSCs. Other pluripotency markers in LD-iNSCs were identified by reverse transcription-polymerase chain reaction (RT-PCR). The RT-PCR analysis indicated that some of the endogenous pluripotency marker genes (*SOX2*, *LIN28* and *TERT*) were expressed at levels similar to those of hiPSCs (Fig. 2B). On the other hand, the expression of endogenous genes *OCT4*, *NANOG*, *REX1* and *LEFTY1* had been silenced in all LD-iNSC lines, in contrast to hiPSCs (Fig. 2B). Therefore, in spite of the induction by five reprogramming factors, LD-iNSCs might be multipotent stem cells and not pluripotent cells. The constitutive growth potential of LD-iNSCs was evaluated by long-term culturing of Dox-free LD-iNSCs. These cells survived regardless of the ectopic expression of reprograming factors for more than 12 weeks, and expressed *TERT*, but not endogenous *OCT4* (Fig. S3A,B). Notably, it is well known that the forced expression of *TERT* inhibits replicative senescence and extends lifespan in numerous cell types (Bodnar et al., 1998). Therefore, our results may also suggest that the expression of *TERT* extends the replicative potential of LD-iNSCs.

We then performed bisulfite-sequencing analysis in the promoter region of the pluripotency markers (*OCT4*, *NANOG* and *REX1*) to determine the levels of DNA methylation in LD-iNSCs. *NANOG* and *REX1* promoters were hypermethylated in all LD-iNSC clones, in contrast to hiPSCs. Despite the absence of *OCT4* expression in LD-iNSCs according to RT-PCR analysis (Fig. 2B), the *OCT4* promoter was unexpectedly hypomethylated in all LD-iNSC clones, in contrast to the parental somatic fibroblasts (Fig. 2C). To precisely quantitate the levels of endogenous *OCT4*, *NANOG* and *REX1* expression, we determined the level of their mRNA expression in LD-iNSCs using quantitative PCR (q-PCR). q-PCR analysis showed that LD-iNSCs failed to reactivate expression of endogenous *OCT4*, *NANOG* and *REX1* (Fig. 2D), similar to the results of the RT-PCR analysis shown in Fig. 2B. Therefore, the repression of *OCT4* expression in LD-iNSCs was not strongly linked to gene silencing, resulting from the methylation of the *OCT4* promoter region. Therefore, to verify the histone modification state in the *OCT4* promoter region, we determined the presence of activating histone markers in the *OCT4* promoter region, which are also known to be regulators of pluripotency (Bernstein et al., 2006). We analyzed the presence of the activating histone marker, histone 3 lysine 4-trimethylation (H3K4me3), in the *OCT4* promoter region using chromatin immunoprecipitation (ChIP) and q-PCR. H3K4me3 enrichment was observed in the *OCT4* promoter region of hiPSCs, but not in LD-iNSC#18 (Fig. 2E). These results suggested that the suppression of *OCT4* mRNA expression in LD-iNSCs was caused by a low level of transcriptionally active H3K4 methylation markers in the *OCT4* promoter region. However, reduced H3K4me3 deposition at active promoters in stem cells does not drastically affect steady-state transcription (Clouaire et al., 2012; Jiang et al., 2011). Therefore, *OCT4* expression in LD-iNSCs might also be regulated by other epigenetic mechanisms and transcription factors.

### LD-iNSCs display characteristics of primitive neural stem cells

**Fig. 3.**
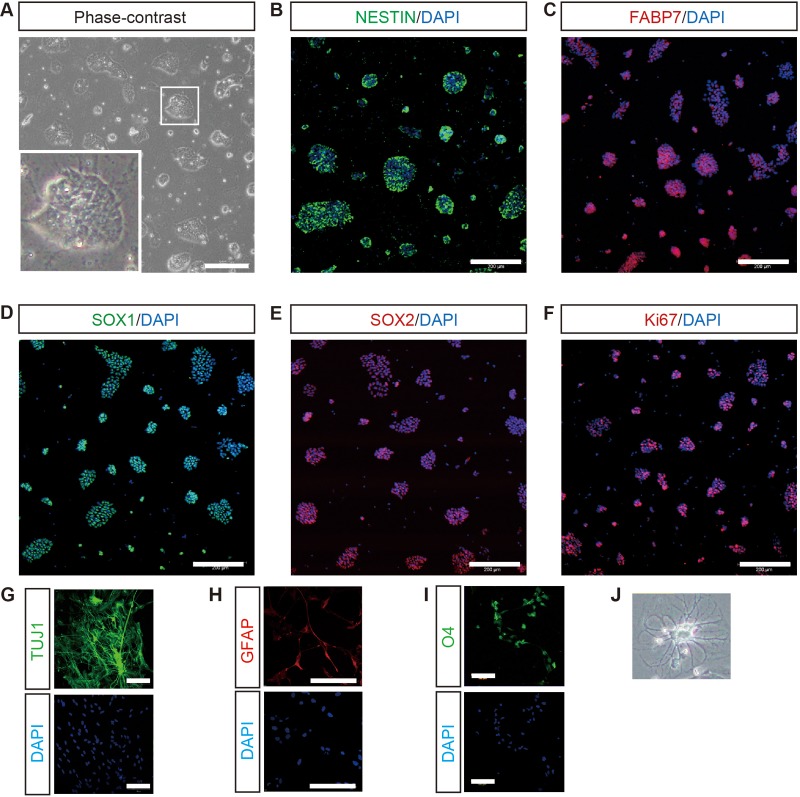
**Expression of neural progenitor markers in LD-iNSCs.** (A) A phase-contrast image of LD-iNSCs cultured onto poly-L-lysine and laminin-coated dishes under feeder-free conditions in the presence of 2i/LIF. A high-magnification image of cells is also shown in the inset image. (B-E) LD-iNSCs without feeder cells in the presence of 2i/LIF tested positive for NESTIN (D, green), FABP7 (E, red), SOX1 (F, green) and SOX2 (G, red). DAPI staining is shown in blue. (F) Immunostaining for Ki67 (red) in LD-iNSCs without feeder cells in the presence of 2i/LIF. DAPI staining is shown in blue. (G-I) An immunofluorescence assay for the detection of markers of neurons (TUJ1), astrocytes (GFAP) and oligodendrocytes (O4). LD-iNSCs have the capacity to differentiate into neurons, astrocytes and oligodendrocytes *in vitro*. (J) Representative image of an oligodendroglial morphology. Scale bars are 200 μm (A-F) or 100 μm (G-I).

We next tested if the LD-iNSCs possessed the characteristics of primitive human NSCs (hNSCs). LD-iNSCs were grown without a feeder layer in the presence of 2i/LIF/Dox in order to verify their morphology. As shown in Fig. 3A, LD-iNSCs displayed a dome-shaped morphology, consistent with previous findings (Theunissen et al., 2014). Immunofluorescence analysis also indicated the expression of NSC markers, including NESTIN, SOX1, SOX2 and FABP7 (Fig. 3B-E). In addition, the LD-iNSCs were highly proliferative, as evidenced by the expression of Ki67 in a majority of the cells (Fig. 3F, Fig. S4A).

Recently, neural stem/progenitor cells have been directly generated from primate fibroblasts by inducing with Yamanaka factors in a chemically defined medium containing LIF, GSK3 inhibitor (CH), and the transforming growth factor (TGF)-β inhibitor SB431542 (SB) (Lu et al., 2013). It is also known that this unique combination of CH, SB and LIF is required for long-term self-renewal of primitive NSCs in a chemically defined culture medium (Li et al., 2011). Therefore, we tested if LD-iNSCs could also grow in the presence of CH/SB/LIF, as well as 2i/LIF. As shown in Fig. S4B, LD-iNSCs showed long-term self-renewal when passaged serially on Matrigel with CH, SB and LIF. We further attempted to confirm the multipotency of LD-iNSCs by evaluating their ability to differentiate into neurons, astrocytes and oligodendrocytes. Immunostaining revealed the appearance of TUJ1-positive neurons, GFAP-positive astrocytes and O4-positive oligodendrocytes (Fig. 3G-J). Likewise, LD-iNSCs generated from BJ foreskin fibroblasts also displayed the ability to differentiate into a lineage of neurons and glia (Fig. S4C), suggesting that LD-iNSCs are indeed multipotent NSCs.

### LD-iNSCs differentiate into neurons *in vitro*

**Fig. 4.**
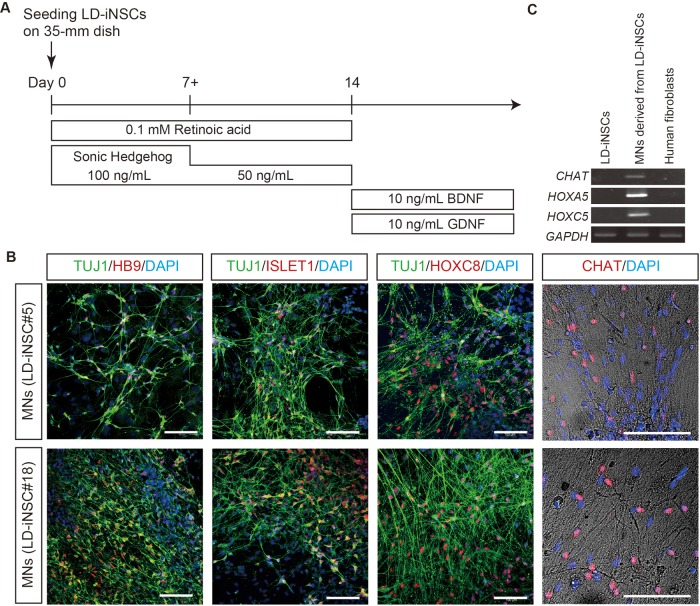
**Differentiation of LD-iNSC into motor neurons.** (A) Schematic representation of the differentiation of LD-iNSCs into motor neurons. (B) LD-iNSC-derived motor neurons were positive for TUJ1 and expressed motor neuron markers such as HB9, ISLET1, HOXC8 and CHAT. Cell nuclei were visualized with DAPI. Scale bars are 100 μm. (C) RT-PCR revealed increased/induced expression of postmitotic markers of motor neurons (CHAT, HOXA5 and HOXC5) in LD-iNSCs-derived motor neurons in contrast to LD-iNSCs and human fibroblasts. GAPDH served as the internal control. Abbreviations: BDNF, brain-derived neurotrophic factor; GDNF, glial cell-derived neurotrophic factor.

The above observations suggest that *in vitro*-expanded LD-iNSCs may possess an identity similar to primitive NSCs. We further tested if LD-iNSCs, like NSCs, can differentiate into a specific neuronal subtype. We first confirmed that LD-iNSCs could differentiate into motor neurons. Under the differentiation conditions shown in Fig. 4A, all LD-iNSC clones (#5, #18 and #42.2) expressed pan-neuronal markers, such as TUJ1; these TUJ1-positive neurons also expressed post-mitotic lineage-specific markers of motor neurons, such as homeobox genes *HB9*, *ISLET1* and *HOXC8*, and the choline acetyltransferase neurotransmitter, *CHAT* (Fig. 4B and Fig. S4D). Motor neuron identity was also confirmed at the transcriptional level by RT-PCR (Fig. 4C). Accordingly, these results indicated that our TUJ1-positive neurons had the properties of mature motor neurons. Thus, LD-iNSCs efficiently differentiated into postmitotic motor neurons via simple induction with sonic hedgehog (SHH) and retinoic acid (RA).

**Fig. 5.**
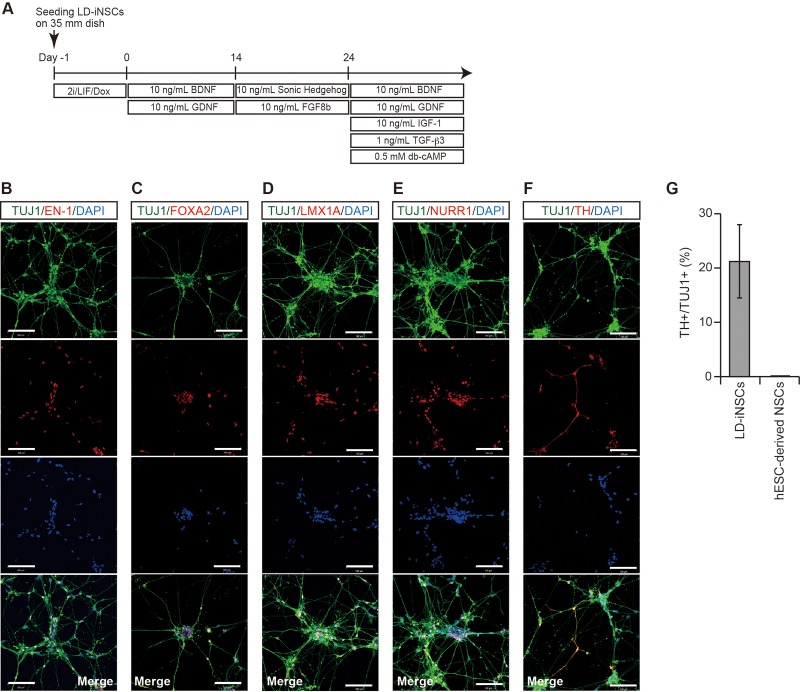
**Differentiation of LD-iNSCs into dopaminergic neurons.** (A) An overview of the protocol for differentiation of LD-iNSCs into dopaminergic neurons. (B-F) Efficient production of dopaminergic neurons from LD-iNSCs was demonstrated by immunostaining for EN-1, FOXA2, LMX1A, NURR1 and TH (red). TUJ1 is a pan-neuronal marker used to assess the total production of neurons (green). Cell nuclei were visualized with DAPI. Scale bars are 100 μm (B-F). Abbreviations: FGF8b, fibroblast growth factor 8 b; IGF-1, insulin-like growth factor-l; TGF-β3, transforming growth factor beta 3; db-cAMP, dibutyryl cyclic adenosine monophosphate. (G) Percentage of TH+ neurons among total neurons (TUJ1+) differentiated from LD-iNSCs and hESC-derived NSCs under DA neuronal differentiation conditions was determined by immunofluorescence. Error bars are mean±s.e.m., *n*=6.

We next tested if LD-iNSCs also have the capacity to differentiate into other neuronal subtypes such as dopaminergic (DA) neurons. The DA neuron differentiation conditions shown in Fig. 5A induced the differentiation of LD-iNSCs into neurons exhibiting EN-1, LMX1A, NURR1 and FOXA2 immunoreactivity (Fig. 5B-E). In addition, these transcription factor genes, encoding for mesencephalic DA neuron markers, were expressed in most of the TUJ1-positive neurons. Tyrosine hydroxylase (TH) was also present in the differentiated LD-iNSCs, suggesting that these cells differentiated into mature dopaminergic neurons. To determine the properties of the LD-iNSCs, we measured the number of DA neurons differentiated from LD-iNSCs and hESC-derived NSCs (*n*=6). The LD-iNSCs yielded a higher proportion of TH+/TUJ1+ DA neurons (21.2±6.7%) than NSCs (negligible) (Fig. 5G). These results indicate that LD-iNSCs have significantly greater potency than hESC-derived NSCs in generating DA neurons.

### Conversion of LD-iNSCs into conventional hiPSCs

Geijsen and colleagues have previously reported that the ability of self-renewal in hLR5 cells is dependent on the expression of ectopic reprogramming factors (OCT4, SOX2, KLF4, c-MYC and NANOG), similar to the LD-iNSCs of this study (Buecker et al., 2010). On the other hand, we do not know if hLR5 cells have the same properties as neural stem/progenitor cells. hLR5 cells can be converted to a stable, epiblast-like pluripotent state by simultaneously removing the ectopic reprogramming factors and altering the culture growth factor conditions. Therefore, we attempted to verify if changes in the growth factor environment could induce a similar conversion of LD-iNSCs to a stable pluripotent state (conventional hiPSCs).

**Fig. 6.**
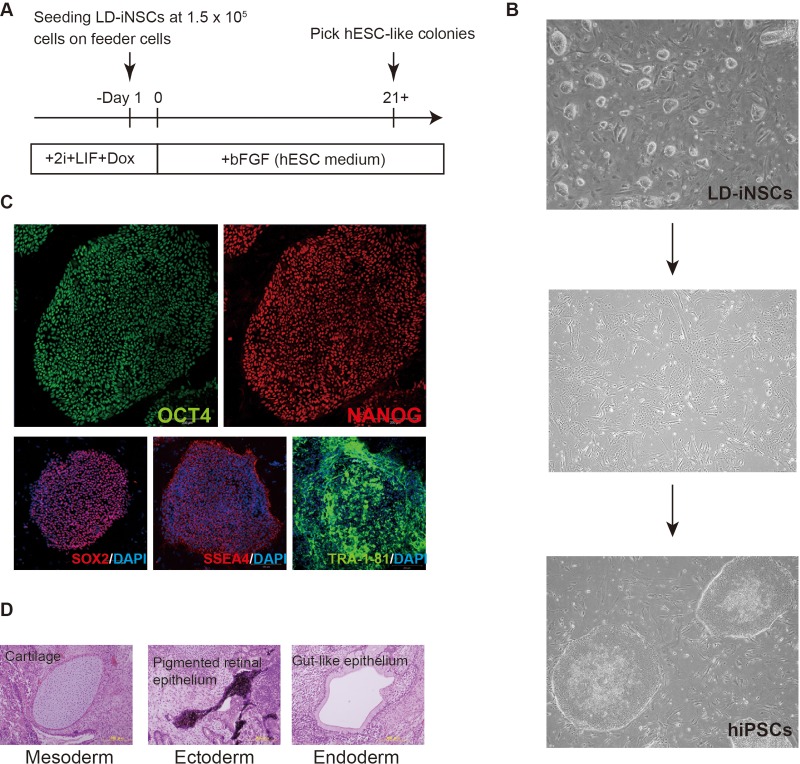
**Conversion of LD-iNSCs to hiPSCs.** (A) Schematic representation of the conversion of LD-iNSCs to a stable pluripotent state. (B) Three days after switching to the standard hESC medium supplemented with bFGF, LD-iNSC lost its colony morphology (top panel) and the cells adopted a fibroblast-like appearance (middle panel). After further growth in this culture condition, LD-iNSCs were converted to hiPSCs (bottom panel). The converted cells (LD5F-hiPSCs) exhibited hESC-like colony morphology. (C) Typical ESC markers (OCT4, NANOG and SOX2) were expressed in LD5-hiPSCs, according to immunofluorescence analysis. Other pluripotent cell surface markers (SSEA4 and TRA-1-81) were also detected by immunofluorescence staining. DAPI was used to visualize the cell nuclei. (D) Injection of undifferentiated LD5F-hiPSCs into immune-deficient mice led to the formation of teratomas containing derivatives of all three germ layers: cartilage (mesoderm, left panel), pigmented retinal epithelium (ectoderm, middle panel) and gut-like epithelium (endoderm, right panel).

Fig. 6A shows the timeline of conversion of LD-iNSCs to conventional hiPSCs. Initially, LD-iNSCs dissociated by trypsinization were plated on feeder cells (density: 1.5×10^5^ cells/100-mm dish) in a medium containing 2i/LIF/Dox. The medium containing 2i/LIF/Dox was switched to hESC medium supplemented with bFGF (Fig. 6A) on day 2. Most colonies of LD-iNSCs immediately broke up after downregulation of the ectopic reprogramming factors (Fig. 6B). Nonetheless, hESC-like colonies emerged on or after day 21, and were mechanically picked for expansion. These colonies eventually displayed a typical hiPSC-like morphology (Fig. 6B). These were therefore referred to as LD-iNSC-derived 5-factor hiPSCs (LD5F-hiPSCs). The conversion frequency of LD-iNSCs into LD5F-hiPSCs was approximately 0.01%, similar to the conversion of hLR5 cells into hiPSCs (Buecker et al., 2010). In order to confirm that the LD5F-hiPSCs (clones #55 and #57) were indeed derived from LD-iNSC #18, we analyzed the clones by Southern blotting for two independent sites in the lentivirus vector, which was randomly integrated into the genome of these cells. The Southern blot revealed that the distribution of all lentiviral vector integration sites in LD5F-hiPSCs was identical to the pattern of the parental LD-iNSC clone #18 (data not shown). Therefore, these results confirmed that LD5F-hiPSCs (clones #55 and #57) were derived from LD-iNSC clone #18. The resulting LD5F-hiPSCs displayed typical hESC morphology, and were subsequently maintained with bFGF alone, indicating that LD5F-hiPSCs need not be activated by the LIF signaling pathway.

**Fig. 7.**
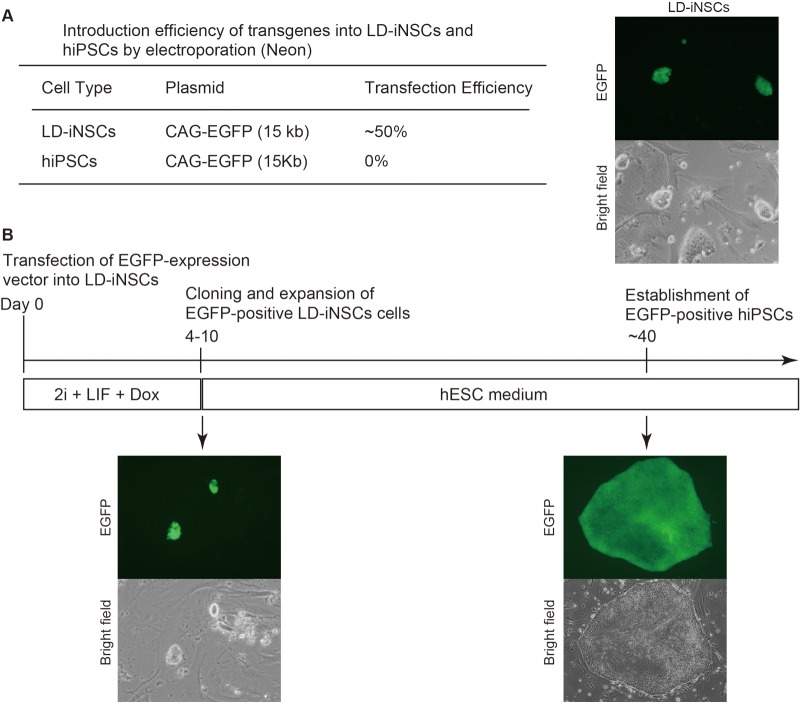
**Introduction of a transgene into LD-iNSCs.** (A) Results of the electroporation experiments with LD-NPs and hiPSCs are summarized in the table (left panel). The right panel shows representative images of LD-iNSCs after electroporation of an EGFP expression vector (15 kb). As shown in the table, the percentage of EGFP-positive LD-iNSC colonies was approximately 50% under our transfection conditions, but negligible or 0% in conventional hiPSCs. (B) Conversion of EGFP-positive LD-iNSCs to a stable pluripotent state. hiPSCs converted from an EGFP-positive LD-iNSC clone were obtained at the indicated time point. mESCs/iPSCs have generally demonstrated a relatively high level of transfection efficiency, whereas hESCs/iPSCs are notoriously difficult to transfect (Cao et al., 2010). Therefore, these results suggest that the conversion to hiPSCs might be a novel method that could increase the transfection efficiency of hiPSCs.

To further characterize the LD5F-hiPSC phenotype, we performed a comprehensive molecular analysis at the protein level. Immunostaining revealed that LD5F-hiPSCs consistently expressed numerous pluripotency markers, including OCT4, SOX2, NANOG, SSEA4 and TRA-1-81 (Fig. 6C). We also attempted to verify if the LD5F-hiPSCs acquired pluripotency by subcutaneously injecting LD5F-hiPSCs into immunosuppressed mice; the resulting tumors were enucleated after 10 weeks. Histological analysis revealed the presence of teratomas containing tissues of all three germ layers, including the cartilage (mesoderm), pigmented retinal epithelium (ectoderm) and gut-like epithelium (endoderm; Fig. 6D). These results indicated that LD5F-hiPSCs had the ability to proliferate indefinitely (without commitment to any cell lineage regardless of the ectopic reprogramming factors) and the capacity to differentiate into cell lineages from the three germ layers. In addition, LD-iNSCs were more amenable to the introduction of transgenes than hiPSCs (Fig. 7A,B), suggesting that LD-iNSCs can be useful in research and the development of novel cell therapies for neurodegenerative diseases.

## DISCUSSION

**Fig. 8.**
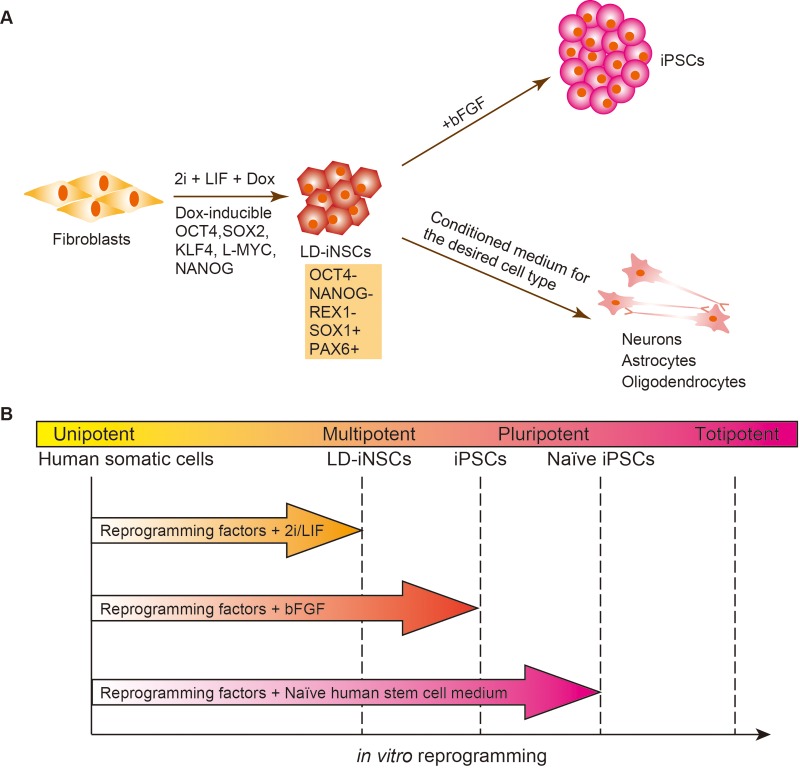
**LIF-dependent primitive NSCs are obtained in the process of reprogramming to iPSCs.** (A) The establishment of various desired neuronal types by means of multipotent LD-iNSCs. LD-iNSCs are directly derived from human fibroblasts via continuous expression of reprogramming factors in the presence of 2i/LIF in the culture medium. LD-iNSCs retain low levels of OCT4 and NANOG expression, but high levels of SOX2 expression, have self-renewal capacity, and can differentiate into various neuronal types (such as motor neurons and dopaminergic neurons), astrocytes and oligodendrocytes. In addition, pluripotent stem cells can be derived by simple re-plating of LD-iNSCs in hESC medium supplemented with bFGF, suggesting that LD-iNSCs are derived from intermediate partially reprogrammed cells. (B) Pluripotency levels induced by *in vitro* reprogramming correlate significantly with the extrinsic signaling environment. The delivery of reprogramming factors into primary human fibroblasts, and culturing of the cells in the presence of 2i/LIF leads to the resulting cell lines (such as LD-iNSCs) resembling multipotent primitive neural stem/progenitor cells (Hirano et al., 2012). Aside from the 2i/LIF treatment, bFGF signaling plays an important role in the derivation and maintenance of hiPSCs (a primed state). Fully naïve hiPSCs were recently derived using a novel medium called naïve human stem cell medium (NHSM) (Gafni et al., 2013). Taken together, the combination of reprogramming factors and culture supplements determines the respective pluripotent states.

In this study, we investigated a strategy for the generation of multipotent stem cells (LD-iNSCs) with NSC properties from human fibroblasts using five reprogramming factors (OCT4, KLF4, SOX2, L-MYC and NANOG). The efficiency of LD-iNSC generation from fibroblasts was approximately 0.01–0.03%, as determined from the initial number of fibroblasts (1.5×10^5^) used in the reprogramming experiments. These LD-iNSCs showed self-renewal capacity in the presence of LIF and inhibitors of GSK3β and ERK1/2 signaling, and five ectopic reprogramming genes. The expanded LD-iNSCs are capable of differentiating into neurons, astrocytes and oligodendrocytes, suggesting that LD-iNSCs have the predictable identity of LIF-dependent primitive NSCs (Fig. 8A). In general, mouse primitive NSCs (LIF dependent, but independent of exogenous bFGF) can be derived from mESCs *in vitro* or from the epiblast [embryonic day 5.5 (E5.5) to E7.5] and the neuroectoderm (E7.5–E8.5) *in vivo* (Tropepe et al., 2001). Definitive NSCs (LIF independent and bFGF dependent) first appear in the E8.5 neural plate and persist for life (Wada et al., 2006). Therefore, LIF-dependent primitive NSCs give rise to definitive NSCs (Hitoshi et al., 2004), suggesting that primitive NSCs have a higher neurogenic potential than definitive NSCs. Therefore, this indicated that LD-iNSCs are capable of differentiating into bFGF-dependent definitive NSCs. However, the replacement of 2i/LIF medium with neural stem cell medium supplemented with bFGF and EGF led to a majority of the cells losing their NSC-like ability, and thereby could not be cultured for more than one month. LIF functions as a survival factor for primitive NSCs (Smukler et al., 2006). Therefore, when LIF is removed from the primitive NSC culture system to induce bFGF-dependent definitive NSCs, the passage of primitive NSCs to definitive NSCs may result in significant cell death. On the other hand, once a population of definitive NSCs is established after LIF withdrawal, these cells can propagated in NSC culture medium supplemented with bFGF. Therefore, this initial adaptation to cell culture conditions may be important for the prevention of cell death. The establishment and long-term maintenance of primitive hNSCs from embryonic, fetal, newborn, and adult tissues is a difficult task; there are also limitations on the number of graft materials that can be used for primitive hNSCs. Primitive hNSCs can also be derived from hESCs/iPSCs; however, chemically defined conditions for the long-term maintenance of primitive hNSCs are unknown (Li et al., 2011). Therefore, the induction of primitive hNSCs from hESCs/iPSCs may result in ineffective generation of hNSCs or retention of hESC/iPSC characteristics. This increases the risk of development of immature teratomas following the transplantation of iPSC-derived multipotent stem cells or their progeny, as a result of possible iPSC contamination or incomplete reprogramming. This suggests that there are several limitations to the use of iPSCs in neural cell-based therapy or in neural disease modeling.

In general, reprogramming factors such as the Yamanaka factors are used to drive somatic cells into a pluripotent state. Kim et al. (2011) showed that mouse fibroblasts are converted to neural stem cells by bypassing the iPSC stage with help from the same factors. Therefore, our study might also support the hypothesis that neural stem cells can be derived during the iPSC induction process.

Unexpectedly, LD-iNSC colonies appeared approximately after day 14 in this study, whereas the iPSC colonies were not observed for 3–4 weeks after viral infection in the presence of 2i/LIF and Dox. These data suggest that LD-iNSCs are most likely generated via direct conversion from fibroblasts rather than through rapid differentiation from iPSCs into primitive NSCs. Our LD-iNSCs were reprogrammed into a stable pluripotent state when the culture medium was changed to hESC medium supplemented with bFGF (Fig. 8A). This finding indicates that LD-iNSCs are derived from intermediate, partially reprogrammed cells. Further characterization of LD-iNSCs showed that the pluripotency genes – *OCT4*, *NANOG* and *REX1* – was not significantly activated during this process. The silenced status of these genes was also confirmed by epigenetic changes in the promoter regions of the relevant pluripotency regulators. The *NANOG* and *REX1* promoter regions were hypermethylated in LD-iNSCs. Unexpectedly, the methylation level in the *OCT4* promoter region in LD-iNSCs was the same as that in hiPSCs. Similarly, the methylation status of the *OCT4* promoter in LIF-dependent primitive NSCs is also low, similar to ESCs (Akamatsu et al., 2009). Therefore, *OCT4* silencing in LD-iNSCs might be attributed to a decrease in the level of the transcriptionally active H3K4 methylation marker, than the promoter methylation status. Hypomethylation of the *OCT4* promoter region indicates that LD-iNSCs are in a state that is poised towards pluripotency. Therefore, the fact that LD-iNSCs are partially reprogrammed intermediate cells could also be explained in terms of the epigenetic status. However, cultivation of stem cells in the presence of 2i (two small molecules: CHIR99021 and PD0325901) and LIF can induce a naïve state in mouse pluripotent stem cells (Hanna et al., 2009; Ying et al., 2008). Moreover, several groups recently generated mESC-like hiPSCs by applying the naïve culture conditions after inducing with reprogramming factors; however, such cell lines lack the phenotypic stability that distinguishes bona fide mESCs/iPSCs (Hanna et al., 2010; Li et al., 2009). In this study, we did not detect the expression of pluripotency markers in several of the hiPSC lines when cultivating the cells in the presence of 2i and LIF, and absence of reprogramming factors. Moreover, none of the examined hiPSC lines differentiated into LIF-dependent primitive neural progenitors in the presence of 2i/LIF alone. This result indicates that the ectopic expression of specific reprogramming factors is essential for the self-renewal of LD-iNSCs. This finding is consistent with those of a previous report, wherein the exogenous reprograming factors were shown to be reactivated in primitive NSCs derived from hiPSCs in the presence of 2i/LIF (Hirano et al., 2012). Similarly, recent publications also suggest that the stability of transdifferentiated phenotypes appears to depend on the sustained overexpression of reprogramming transgenes (Ieda et al., 2010; Sheng et al., 2012). Li et al. (2011) have reported that the long-term self-renewal of hESC-derived neural stem cells is sustained by a chemically defined medium supplemented with LIF, CHIR99021 and SB431542 (TGF-β inhibitor). Thus, these findings suggest that the GSK3β and TGF-β pathways are not essential for the self-renewal of LIF-dependent primitive NSCs. This evidence is in line with our data, which showed that the colonies of LD-iNSCs also grow in medium supplemented with LIF, CHIR99021 and SB431542 (Fig. S4B). In our study, LD-iNSCs colonies were formed in the presence of PD0325901 (inhibitor of ERK1/2 phosphorylation) instead of SB431542. This observation suggested that 2i/LIF promotes the conversion of human fibroblasts to primitive NSCs when accompanied by ectopic expression of reprogramming factors, but does not elicit a self-renewal response in hiPSCs. Gafni et al. (2013) recently showed that naïve pluripotent stem cells can be established in humans using a unique combination of cytokines and small molecule inhibitors in addition to 2i/LIF; this medium, termed naïve human stem cell medium (NHSM; Fig. 8B), further supported the concept that culture environments could control the fate of reprogrammed cells (Han et al., 2011). As described above, LD-iNSCs may metastably settle into a state poised toward pluripotency, suggesting that the replacement of 2i/LIF with NHSM may elevate LD-iNSCs to a more pluripotent status from the standpoint of the hierarchy of pluripotency.

In summary, we successfully developed methods for the direct generation of LIF-dependent primitive iNSCs from human fibroblasts in the presence of 2i/LIF. Our methods may be applied to cells isolated from patients with neurological disorders, such as Parkinson's disease, amyotrophic lateral sclerosis, Alzheimer's disease, or other neurodegenerative diseases. These patient-specific LD-iNSCs should be useful in future drug screening and for the modeling certain neurological diseases.

LD-iNSCs can be stably expanded for more than 100 passages in the presence of 2i/LIF/Dox, suggesting that the plasticity of the LD-iNSCs is maintained in the long run. In the future, LD-iNSCs may also turn out to be useful for cell therapy, for e.g. patients with spinal cord injury. In conclusion, this study might open the possibility of generating LD-iNSCs from patients for cell therapy and for identification of drug targets in neurological diseases.

## MATERIALS AND METHODS

### Ethics statement

Primary human fibroblasts were used with approval from the Institutional Review Board of the National Institute for Child Health and Development, Japan. Signed informed consent forms were obtained from the donors, and the surgical specimens were irreversibly de-identified. All experiments that involved handling of human cells and tissues were performed in line with the tenets of the Declaration of Helsinki.

### Animal experiments

Research involving animals complied with the protocols approved by the Institutional Animal Care and Use Committee of the National Research Institute for Child Health and Development.

### Generation of LD-iNSCs

LD-iNSCs were produced using the following procedure. BJ foreskin fibroblasts were purchased from American Type Culture Collection (ATCC) – alternatively, primary human fibroblast-like cells from menstrual blood were isolated as described previously (Cui et al., 2007) – and grown in 10% fetal bovine serum (FBS)/Dulbecco's modified Eagle medium (DMEM); the cells displayed a spindle*-*shaped morphology. Primary human fibroblasts (1.5×10^5^) were plated in a 6-well plate; after 24 h, two lentiviral vectors (carrying doxycycline-inducible TRE3G promoter-driven human *OCT4*, *KLF4*, *SOX2*, *L-MYC* and *NANOG* and the CAG promoter-driven Tet-On 3G transactivator) were transduced into the cells. Three days after the transduction, primary human fibroblasts were passaged by trypsinization, and re-plated on irradiated mouse embryonic fibroblast (MEF) feeder layers. LD-iNSCs were induced by replacing the medium with LD-iNSC induction medium supplemented with 0.5× Knockout DMEM/F12 medium (Life Technologies, Carlsbad, CA, USA), 0.5× Knockout Neurobasal Medium (Life Technologies), 0.5× B27 (Life Technologies), 0.5× N2 supplement (Life Technologies), 10 μg/ml recombinant human insulin (Wako Pure Chemical Industries, Osaka, Japan), 0.1% recombinant human LIF (Wako Pure Chemical Industries), 0.005% bovine albumin fraction V (Life Technologies), 1% GlutaMAX (Life Technologies), 1% nonessential amino acids (Life Technologies), 55 nM β-mercaptoethanol (Life Technologies), 100 U/ml penicillin-streptomycin (Life Technologies), 2% KnockOut Serum Replacement (Life Technologies), and 64 µg/ml L-ascorbic acid-2-phosphate magnesium (Sigma-Aldrich, St. Louis, MO, USA). Neural progenitor-like round-shaped colonies usually appeared after 14–28 days. The colonies were picked, seeded in individual wells, and cultured on MEF feeder layers with the LD-iNSC induction medium supplemented with two small molecule inhibitors (2i): PD0325901 (1 µM, ERK1/2 inhibitor; Wako Pure Chemical Industries) and CHIR99021 (3 µM, GSK3β inhibitor; Axon Medchem, Fairfax, VA, USA). The cells were seeded at >2×10^5^ cells per 60-mm dish using Accutase (Life Technologies) every 3–4 days, depending on the culture density. LD-iNSCs and hiPSCs cells were collected using Accutase, and resuspended in buffer R (provided with the *Neon* Electroporation System; Life Technologies) for transfection; the resuspended cells were transfected with 3 μg of the DNA constructs using the Neon Transfection System (Life Technologies; 1400 V, 10 ms, 3 pulses for LD-iNSCs; 1050 V, 30 ms, 2 pulses for hiPSCs).

Although the LD-iNSCs established in this study were successfully maintained as described above, LD-iNSCs could also be grown in Geltrex-coated plates for at least one month. Moreover, according to the conventional culture conditions for primitive NSCs, LD-iNSC induction medium supplemented with 3 µM CHIR99021 and 2 µM SB431542 (TGF-β inhibitor; Tocris Bioscience, Bristol, UK) under feeder-free (Matrigel) culture conditions (Li et al., 2011) was also used.

### Derivation of hiPSCs

LD-iNSCs were plated at a density of 1.5×10^5^ cells per 100-mm dish on MEF feeder layers in LD-iNSC induction medium, in the presence of 2i/LIF/Dox. Dox was removed from the medium the next day, and the cells were further maintained in hESC medium containing Knockout DMEM supplemented with 20% Knockout serum replacement, 10 ng/ml recombinant human bFGF (Life Technologies), 1% GlutaMAX, 1% nonessential amino acids, 55 nM β-mercaptoethanol, 1 mM sodium pyruvate (Life Technologies), and 100 U/ml penicillin-streptomycin. Typical hiPSC-like colonies emerged after approximately 3 weeks, and were mechanically picked and seeded on fresh MEF feeder layers. The colonies were passaged mechanically every 5–7 days.

### Neural differentiation *in vitro*

As shown in Figs 4A and 5A, the protocol for *in vitro* differentiation of LD-iNSCs into motor neurons or dopaminergic neurons was modified from a previously reported method (Li et al., 2011). The development of motor neurons can be recapitulated *in vitro* by the addition of RA and an SHH agonist (Hh-Ag1.3) to the culture medium of ESCs (Wichterle et al., 2002). Based on this evidence, we used the procedure shown in Fig. 4A to differentiate LD-iNSCs into motor neurons. LD-iNSCs were plated on dishes coated with laminin/poly-L-ornithine, and treated with 0.1 mM RA and SHH (100 ng/ml) for 7 days. These cells were then cultured in the presence of 0.1 mM RA and the SHH (50 ng/ml) for 7 days, and finally cultured in the differentiation medium supplemented with 10 ng/ml brain-derived neurotrophic factor (BDNF) and 10 ng/ml glial cell-derived neurotrophic factor (GDNF). The protocol shown in Fig. 5A was used for DA neuron differentiation. In this protocol, LD-iNSCs or hESC (H9)-derived NSCs (Life Technologies) were first treated with 10 ng/ml BDNF and 10 ng/ml GDNF for two weeks, and subsequently with 100 ng/ml SHH and 100 ng/ml FGF8b for 10 days. Finally, these cells were further differentiated in the presence of 10 ng/ml BDNF, 10 ng/ml GDNF, 10 ng/ml insulin-like growth factor (IGF)-1, 1.0 ng/ml TGF-β3, and 0.5 mM dibutyryl-cAMP (dbcAMP) for another 2–3 weeks.

### RNA expression analysis

RNA was isolated from sub-confluent growing cells using the RNeasy Mini Kit (Qiagen, Venlo, Netherlands), and DNA was removed using DNase (Life Technologies) to avoid genomic DNA amplification. First-strand cDNA was synthesized from 2 μg RNA using SuperScript III reverse transcriptase and an oligo-dT primer (Life Technologies) according to the manufacturer protocols. PCR reactions were performed using TaKaRa Ex Taq Hot Start (Takara Bio Inc., Shiga, Japan). The PCR primer sequences are summarized in Table S2. All PCR products were analyzed by 2% agarose gel electrophoresis, staining with ethidium bromide, and visualizing the stained gels under ultraviolet light. Quantitative PCR (q-PCR) was performed in triplicate on a QuantStudio 12K Flex Real-Time PCR System (Applied Biosystems, Waltham, MA, USA) using the SYBR Green PCR Master mix (Applied Biosystems). Dissociation curves were constructed after amplification to ensure the amplification of each PCR product. All primer sequences are summarized in Table S2. QuantStudio 12K Flex Software v1.0 was used to quantify the expression of each mRNA segment. Glyceraldehyde 3-phosphate dehydrogenase (*GAPDH*), a housekeeping control gene, was used for normalization.

### Alkaline phosphatase staining and immunofluorescence analysis

Alkaline phosphatase activity was determined using the BCIP/NBT Substrate System (Dako, Glostrup, Denmark). For immunofluorescent staining, the cells were fixed with 4% paraformaldehyde in phosphate-buffered saline (PBS; Wako Pure Chemical Industries) for 5 min at 4°C, and permeabilized with 0.2% Triton X-100 for 2 min at room temperature. The fixed cells were blocked with a normal serum solution (Dako) at room temperature. The following primary antibodies were used: mouse anti-Oct4 (Santa Cruz Biotechnology, Santa Cruz, CA, USA), mouse anti-Nanog (ReproCELL, Yokohama, Japan), rabbit anti-Sox2 (Merck-Millipore, Darmstadt, Germany), mouse anti-SSEA4 (Millipore), mouse anti-TRA-1-81 (Millipore), rabbit anti-Sox1 (Abcam, Cambridge, UK), rabbit anti-Pax6 (Abcam), rabbit anti-Nestin (Sigma-Aldrich), rabbit anti-BLBP (Millipore), mouse anti-Ki-67 (BD Pharmingen, Franklin Lakes, NJ), mouse anti-βIII tubulin (Promega Corp., Madison, WI, USA), rabbit anti-GFAP (Sigma-Aldrich), mouse anti-O4 (Merck-Millipore), rabbit anti-GFAP (DAKO), rabbit anti-HB9 (Abcam), rabbit anti-Islet1 (Abcam), rabbit anti-HoxC8 (Abcam), goat anti-ChAT (Merck-Millipore), rabbit anti-EN-1 (Santa Cruz Biotechnology), rabbit anti-FoxA2 (Merck-Millipore), rabbit anti-Lmx1 (Merck-Millipore), rabbit anti-Nurr1 (Santa Cruz Biotechnology), and rabbit anti-tyrosine hydroxylase (Merck-Millipore). The day after incubation at 4°C with a primary antibody, the cells were incubated with the appropriate secondary antibody (Life Technologies) for 60 min at room temperature. The cell nuclei were counterstained with DAPI (0.5 mg/ml). The staining was visualized using a laser scanning confocal microscope (Carl Zeiss).

### Teratoma formation assay

hiPSCs were suspended at 1×10^7^/ml in PBS. The cell suspension (100 μl, 10^6^ cells) was injected subcutaneously into the dorsal flank of nude mice (Clea Japan, Tokyo, Japan). Two to three months after the injection, the tumors were surgically excised from the mice. The tumor specimens were fixed in PBS containing 4% formaldehyde, and embedded in paraffin. The sections were histologically examined after hematoxylin and eosin staining.

### ChIP q-PCR

Feeder-free iPS cells (2×10^7^) were harvested by trypsinization and fixed in formaldehyde (final concentration 1%). The formaldehyde-fixed cells (5×10^6^) were resuspended in NP-40 lysis buffer (ChIP Reagent; Nippon Gene Co., Ltd., Tokyo, Japan) containing a 1× protease inhibitor mix (P.I.; Nippon Gene Co., Ltd.), mixed well by vortexing, and incubated on ice for 10 min. The cells were then resuspended in sodium dodecyl sulfate (SDS) lysis buffer (ChIP Reagent; Nippon Gene Co., Ltd.) and the lysate was sonicated to fragment chromatin using a Covaris S220 focused-ultrasonicator. The chromatin was purified by centrifugation and immunoprecipitated with protein A-beads (Veritas Prep., Malibu, CA, USA) conjugated to an anti-H3K4me3 antibody (Millipore) or rabbit IgG (Abcam) in Buffer A with P.I. (LowCell ChIP kit; Diagenode, Liege, Belgium) overnight at 4°C. The chromatin-containing beads were washed with Buffers A and C (LowCell ChIP kit). The washed chromatin-containing beads were incubated in ChIP direct elution buffer (ChIP Reagent) for 20 min at 95°C, and then incubated with proteinase K for 2 h at 55°C. DNA immunoprecipitated from the supernatant was purified using Agencourt AMPure XP beads (Beckman Coulter, Pasadena, CA, USA) according to the manufacturer protocols. The samples were subjected to q-PCR using SYBR Green (Life Technologies). The primer sequences are summarized in Table S2.

### Bisulfite sequencing

Genomic DNA was isolated from 10^2^–10^7^ cells using the NucleoSpin Tissue Kit (Macherey-Nagel, Duren, Germany) and 200–500 ng of the isolated DNA was further treated with bisulfite (EZ DNA Methylation-Gold Kit; Zymo Research, Irvine, CA, USA), according to instructions provided by the manufacturers. The bisulfite treatment converted all unmethylated cytosine bases to uracil, while methylated cytosine remained unchanged. The promoter regions of *OCT4*, *REX1* and *NANOG* were amplified by PCR for bisulfite DNA sequencing, using primers listed in Table S2. PCR conditions were as follows: 35–40 cycles of denaturation at 94°C for 30 s, annealing at 58°C for 30 s, and extension at 72°C for 60 s, using the KAPA HiFi HotStart Uracil+ kit (Kapa Biosystems, Wilmington, MA, USA). The amplified DNA product (with the correct size) was isolated by 2% agarose gel electrophoresis, purified using a QIAquick Gel Extraction kit (Qiagen), and ligated into the pGEM-T easy vector (Promega Corp.). More than 10 clones were randomly picked per cell sample for sequencing. A detailed profile of the DNA methylation sites was analyzed using QUMA (Kumaki et al., 2008).

### DNA/RNA fluorescence *in situ* hybridization (FISH)

Cells were harvested using Accutase (Life Technologies); a single-cell suspension was prepared and plated on vitronectin-coated chamber slides. The cells grown on chamber slides were treated with 0.005% pepsin/0.1 N HCl for 3 min, and fixed in 4% paraformaldehyde for 30 min. cDNA probes specific to the XIST exon 1 and 6 regions were generated for XIST detection; these were labeled by nick translation using the Cy3-labeled dUTP. The HXO-10 probe (Chromosome Science Labo Inc., Sapporo, Japan) was used for the detection of X chromosome. The reaction mixtures containing the XIST DNA and HXO-10 probes were combined for RNA FISH, and added to the cells on the slide. The probes were carefully covered with a coverslip and incubated overnight. The slides were subsequently washed, and a DAPI mounting medium was applied to each cell spot. FISH signals were visualized using a Leica DMRA2 microscope (Leica, Wetzlar, Germany). Images were acquired using CW4000-FISH (Leica). All FISH analyses were performed commercially by Chromosome Science Labo Inc.
